# Study protocol: An investigation of mother-infant signalling during breastfeeding using a randomised trial to test the effectiveness of breastfeeding relaxation therapy on maternal psychological state, breast milk production and infant behaviour and growth

**DOI:** 10.1186/s13006-017-0124-y

**Published:** 2017-07-14

**Authors:** N.H. M. Shukri, J. Wells, F. Mukhtar, M.H.S. Lee, M. Fewtrell

**Affiliations:** 1 UCL Great Ormond Street Institute of Child Health, University College London, WC1N 1EH, London, UK; 2 Faculty of Medicine & Health Sciences, Universiti Putra Malaysia, Seri Kembangan, Malaysia; 3 Department of Nutrition & Dietetics, Faculty of Medicine & Health Sciences, Universiti Putra Malaysia, 43400 UPM, Serdang, Malaysia; 4 Department of Psychiatry, Faculty of Medicine & Health Sciences, Universiti Putra Malaysia, 43400 UPM, Serdang, Malaysia

**Keywords:** Breastfeeding, Mother-infant signalling, Infant growth, Infant behaviour, Maternal stress, Relaxation therapy

## Abstract

**Background:**

The physiological and psychological signalling between mother and infant during lactation is one of the prominent mother-infant factors that may influence breastfeeding outcomes. The infant can ‘signal’ his needs through vocalisation, and the mother can respond by allowing or restricting nipple access, which might alter the breast milk composition or volume. This may lead to parent-offspring conflict during the lactation period. Challenging infant behaviour has also been associated with maternal psychological distress, which might affect breastfeeding performance. Most attempts to improve breastfeeding rates focus on providing additional support, yet many aspects of the breastfeeding process are poorly understood. Thus, our objective is to investigate mother-infant signalling during breastfeeding by manipulating maternal psychological state using a relaxation therapy intervention. The study will test the hypothesis that mothers who listen to the therapy will be more relaxed/less stressed and this will favourably alter breast milk composition and/or affect milk volume and hence influence infant outcomes.

**Methods:**

A randomised controlled trial will be conducted in first-time breastfeeding mothers and their new-born infants. Pregnant mothers will be recruited at antenatal clinics in Selangor, Malaysia, and four home visits will be carried out at 2, 6, 12 and 14 weeks postnatally. Participants will be randomised into a control and an intervention group in the early post-partum period. Mothers from the intervention group will be asked to listen daily to an audio recording with relaxation therapy during breastfeeding. Maternal psychological state, breastfeeding practices and infant behaviour will be assessed using validated questionnaires. Milk volume will be measured using stable isotopes. Breast milk samples will be collected to measure macronutrient content and hormone levels. Anthropometric measurements (weight, length and head circumference) will be performed during all home visits, including body composition at week 14.

**Discussion:**

The main outcomes will be the effect of the intervention on maternal psychological state, milk production, cortisol levels, and infant behaviour and growth. Secondary outcomes will be associations between breast milk composition and infant appetite and growth. This study aims to provide a greater understanding of maternal-infant factors which influence breastfeeding outcomes and which may be useful targets for future interventions.

**Trial registration:**

ClinicalTrials.gov identifier: NCT01971216.

## Background

Mother-infant signalling involves communication and interaction between a mother and her infant. The factors involved in mother-infant signalling in early infancy can be broadly categorised as psychosocial or physiological. The psychosocial aspects such as physical contact, maternal psychological state and infant behaviour occur in all mother-infant pairs, regardless of their feeding method. However, physiological mechanisms are unique to breastfed infants and their mothers because during breastfeeding, milk synthesis and milk composition do not solely reflect maternal physiological and psychological processes. Breastfeeding also represents a complex physiological and behavioural negotiation between the mother and the offspring [1, 2]. For example, the infant can ‘signal’ his needs through vocalisation or non-nutritive suckling, and the mother can respond by allowing/restricting nipple access, hence down- or up-regulating milk synthesis and affecting milk volume. She can also alter the breast milk composition resulting in different concentrations of macronutrients or bioactive factors. This may result in a competition for energy between a mother and her infant during the lactation period, which is described as parent-offspring conflict. Thus, breastfeeding should not be regarded as a one-way communication or an inevitably harmonious cooperation between the mother and the infant, but rather as a dynamic process which involves complex psychosocial and physiological signalling between both parties [1].

This relationship between infant behaviour and maternal response in terms of breastfeeding behaviour or the composition of milk, particularly bioactive factors (e.g. hormones) in breast milk, is largely unexplored in humans. Moreover, all of these factors are complex and inter-related. Therefore, there is a need for research to explore the non-nutrient components in breast milk that may act as messengers in breastfed infants, potentially affecting the infant’s behaviour and metabolic regulation. For example, infant behaviour and temperament in early life may be affected by the concentrations of certain hormones or endogenous opiates such as cortisol and endorphins [3, 4]. Naturally occurring opiate substances in breast milk such as ß-casomorphins [5] may act as sedatives, and hence blunt or lower infant appetite, whereas ghrelin and leptin may influence feeding behaviour (affecting appetite and satiety) and are suggested to be major factors involved in metabolic regulation in breastfed infants [6]. Therefore, studies have hypothesised that mothers have the potential to shape infant behaviour in early life by the transmission of biologically active compounds including cortisol in milk during breastfeeding [3, 7, 8]. Other studies also suggest that breast milk hormones may influence infant appetite and feeding pattern, which can influence growth and body composition in early and later life [9, 10]. Therefore, to address this, as part of this trial, we will investigate breast milk hormones (cortisol, ghrelin and leptin concentrations) in relation to infant behaviour and growth in early infancy.

Although there are many interesting and unexplored issues in the signalling between mother and infant during breastfeeding, the complexity of the inter-relationships between factors makes it problematic to define cause and effect using an observational study design. Thus, many aspects of the breastfeeding process remain poorly understood, and most attempts to improve breastfeeding rates mainly focus on providing additional support, for example improving maternity leave or providing BF support via groups and organisations, rather than on understanding maternal and infant factors in early life. Therefore, to explore these factors in early human life, we plan to use an experimental approach to investigate causal relationships between maternal psychological state, manipulated using a randomised relaxation intervention, and breast milk volume and composition and infant behaviour and growth. We hypothesise that maternal psychological state is associated with breast milk production which will have consequent effects on infant feeding behaviour and growth. This is because emotional distress in mothers has been reported to inhibit the let-down reflex leading to disruption of milk flow and reduced milk volume [11, 12]. Conversely, milk ejection can be improved by relaxation therapy, as demonstrated in previous studies using relaxation techniques such as guided-imagery and music therapy. Randomised studies in mothers of premature infants found that mothers who listened to guided relaxation/imagery recordings (as a relaxation therapy) produced significantly more milk than control groups [13, 14], but to our knowledge this type of intervention has not been formally tested in mothers who are breastfeeding their healthy term infant, and none of the previous studies investigate the consequent effects on infant outcomes.

In summary, this study aims to investigate the signalling between mother and infant in early life, using an experimental approach to determine causal relationships between maternal psychological state and milk intake, milk cortisol levels, and the consequences for infant behaviour and growth. Other mother-infant factors such as changes in milk leptin, ghrelin and macronutrient concentrations within a feed, infant appetite and temperament will be studied as secondary outcome measures. The results may identify modifiable factors which can be used to encourage and support breastfeeding, including increasing breastfeeding rates. The study, named the Mother-Offspring Milk (MOM) Study, is intended to fill current research gaps by investigating both psychological and physiological mother-infant factors during breastfeeding and by using a more robust methodological design. This manuscript describes the rationale and design of the MOM Study.

## Methods/Design

### Study design

This is a randomised controlled trial that involves first-time healthy breastfeeding (BF) mothers and their full-term infants (*n* = 64 mother-infant dyads). The objective is to identify causal effects of maternal psychological state, manipulated using a relaxation intervention on breast milk volume and composition and infant outcomes (behaviour and growth). In general, this study investigates the mother-infant signalling during breastfeeding and aims to provide a greater understanding of maternal-infant factors which influence the breastfeeding outcomes such as milk yield or milk energy, and which may be useful targets for future interventions.

### Hypotheses and outcome measures

Primary hypotheses: The use of a relaxation tape by breastfeeding mothers starting at week 2 postpartum will result in:

i. reduced maternal stress and anxiety
ii. the production of a higher volume of breast milk
iii. lower milk cortisol concentrations
iv. favourable effects on infant behaviour (less crying, more sleeping)
v. differences in infant body composition

Measures (i) - (v) will be assessed at baseline (week 2) and at 12 weeks in both control and intervention groups to ascertain the long-term effects of the relaxation therapy. Measures of (iii) will also be assessed pre and post a single breast-feed in both groups at 2, 6 and 12 weeks to ascertain the short-term effects of the relaxation therapy. The primary comparison will be the values at 12 weeks adjusted for the baseline value, compared between the two groups. Measures of body composition (v) will also be assessed at 14–16 weeks postnatally.

The primary outcomes will be assessed as follows:

i) maternal stress and anxiety assessed using the Cohen’s Perceived Stress Scale (PSS) and Beck Anxiety Inventory (BAI) respectively (at 12 weeks)
ii) breast milk volume at 12 weeks assessed non-invasively using stable isotope techniques
iii) breast milk cortisol concentrations at 12 weeks
iv) infant behaviour measured using a 3-day diary at 6 weeks
v) infant weight and body composition measured using stable isotopes at 12 weeks

changes in maternal saliva cortisol, breast milk cortisol and milk volume before and after a breastfeeding session at 2, 6 and 12 weeks.

b) Secondary hypotheses:

i. Infant temperament, behaviour and gender influence milk intake and hence early growth and body composition.
ii. Milk composition including non-nutrient factors in breast milk (hormonal constituents; ghrelin and leptin) influence infant appetite and feeding patterns and hence infant growth and body composition

The secondary outcome measures will be:

i. non-nutrient factors in breast milk – leptin and ghrelin
ii. macronutrient composition and milk energy (fat, carbohydrate, protein and total calorie)
iii. infant temperament measured using the Revised-Infant Behaviour Questionnaire (R-IBQ)
iv. infant appetite assessed using the Baby Eating Behaviour Questionnaire (BEBQ)
v. maternal depression assessed using Edinburgh Postnatal Depression Scale (EPDS

### Sample size and study population

The target sample population will be first-time mothers and their newborn infants living in Klang-Valley, Malaysia. The conventional formula [15] for two sample t-test is used to determine the number of infants required to detect a difference between two groups; control and intervention:

$$ \mathrm{n}=16\ \left({\mathrm{SD}}^2/{\mathrm{D}}^2\right) $$

where *n* = number per group, *SD* = standard deviation, *D* = difference between groups.

Hence, a sample of 56 infants (28 per randomised group) would allow the detection of a 0.76 SD difference in milk volume between groups at 80% power with a significance level of α = 0.05 (D = 0.76, SD = 1); this is a biologically plausible difference based on previous studies of the effect of relaxation interventions on milk volume production between control and intervention groups of mothers with preterm infants [13]. Since this study will involve mothers with healthy full-term infants, the effect size may be smaller than the previous study, as mothers with preterm infants are likely to be more stressed. Therefore, to allow for a smaller effect size as well as for drop-outs or failed measurements, it is planned that 80–100 pregnant women will be recruited.

### Recruitment

Recruitment will be performed at selected antenatal clinics in Selangor, where pregnant mothers in the second or third trimester of pregnancy will be approached. Mothers who are planning to exclusively breastfeed and interested in participating in the study will be given an information sheet, and the practical details will be explained. If they are still interested, screening questions will be asked to determine if they meet the eligibility criteria (see below). If participants meet the eligibility criteria, written informed consent will be obtained and they will be enrolled into the study. Contact details will be obtained from them, including their estimated day of delivery (EDD). Mothers will be contacted by the researcher 3 to 7 days after their EDD to check if they have delivered their baby, are breastfeeding and still willing to participate in the study. All participants were also provided with the MOM Study’s contact information if they would like to inform about their birth. At this stage, all participants will be screened again to assess the eligibility criteria for both the mother and baby. If they are eligible and still interested in participating in the study, a home visit will be arranged. Mothers will also be advised that if for any reason and at any time they do not wish to be contacted, they can inform the researcher. All participants will also be offered a manual breast pump (Philips Avent brand) as a token of appreciation.

Advertisements about the study will also be posted on parenting and nutrition websites including organizations’ website such as the Malaysia Breastfeeding Peer Counsellor Group, Nutrition Society of Malaysia and Malaysia Dietetic Association. Flyers and posters will be displayed in places commonly visited by mothers such as private antenatal clinics or hospitals, nursing rooms or baby care rooms at shopping malls or restaurants, health care centres and childcare shops in Selangor, Malaysia. Table 1 shows the eligibility criteria for mothers and infants.

**Table 1:** Eligibility criteria for mother and infant

First screening	Inclusion criteria	Exclusion criteria
Mother (during pregnancy)	Primiparous mother with singleton pregnancy	Multiparous mother or mother of twin pregnancy
	Free from serious illness/chronic disease.	Taking medication for illness or chronic disease.
	Non-smoker	Smoker
	Understands Malay or English	Does not understand Malay or English
Second screening		
Mother (after birth) Infant	Free of illness that can affect breastfeeding	Mother has illness that prevented her from breastfeeding
	Exclusively breastfeeding at 2 weeks	Mixed- or not breastfeeding at 2 weeks
	Interested in participating further	Not interested or unwilling to arrange home visit
	Full-term infant (37–42 weeks of gestation)	Preterm infant (<37 weeks of gestation)
	Infant birth weight of ≥2500 g	Infant birth weight of <2500 g
	Free from serious illness that could affect nursing or growth	Has illness that could affect nursing or growth

### Data collection

**Fig. 1:**
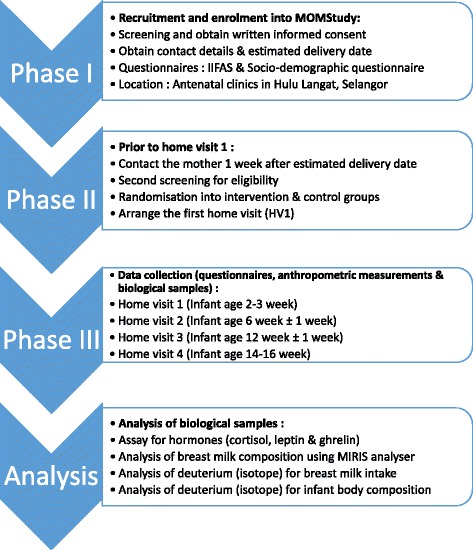
Overview of the MOM Study plan

**Fig. 2:**
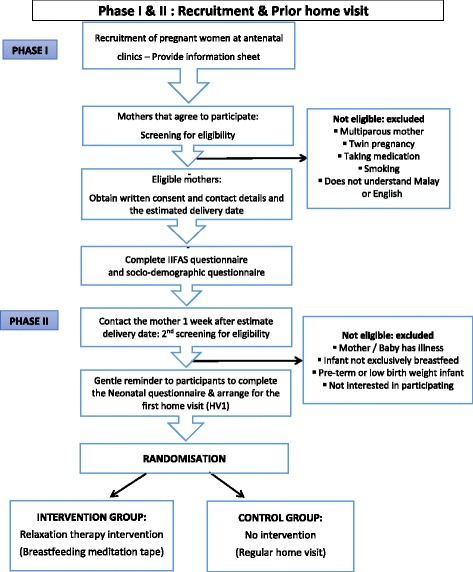
Recruitment, enrolment and randomisation process

**Fig. 3:**
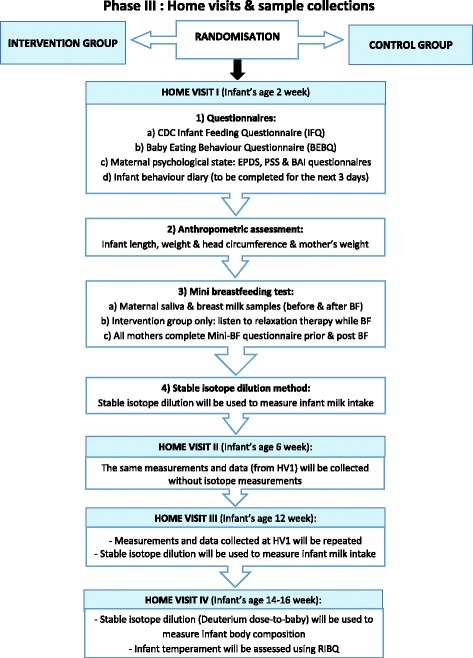
Data collection processes and procedures

In this section, the process of data collection is explained first, including the tools used for measurement and assessment. Next, a detailed description of each tool and measurement is provided, including information about the development of the intervention tool. Research flow charts illustrating the overview plan of the study and the overall data and sample collection procedures are shown in Figs. 1, 2 and 3.

#### Post-enrolment session

Once signed consent has been obtained, participants will be provided with questionnaires to be completed immediately following enrolment at the antenatal clinic, namely the socio--demographic questionnaire and Iowa Infant Feeding Attitude Scale (IIFAS) questionnaire.

#### Randomisation

Prior to the first home visit (at 2–3 weeks after delivery), and after confirming that the mother is still eligible and wishes to continue in the study, the mother will be randomised to either the control group or the relaxation group. Randomisation will be stratified by ethnicity: Malay or Bumiputera, Chinese and Indian. Mothers will not be informed about this process as doing so will most likely influence their behaviour. Thus, the mothers will be blinded to the randomisation process in order to prevent the mothers in the control group from seeking or using some form of relaxation therapy, if the possibility of it being beneficial has been raised. However, they will be informed that the MOM Study is investigating the effects of maternal mood as well as infant factors on breastfeeding at a general level. The randomisation schedule will be generated by computer in randomised blocks of permuted length (2,4,6). Assignments will be prepared by a member of the research team in London who does not have contact with the subjects and assignments will be held in sealed opaque envelopes. Each assignment will be revealed by the researcher on the day, just before the first home visit, in order to prepare the intervention materials including the diary log specifically for mothers in the intervention group. There will be a low possibility of contamination between randomised groups since home visit sessions will be performed over a large geographical area in the central region of Malaysia. Therefore, the mothers in the intervention group will not be likely to have the opportunity to reveal the existence of the intervention tool to mothers in the control group. All mothers will only be informed about the randomisation process when the trial has been completed, when they receive a summary of the results.

#### Postnatal Home visits (HV1–3)

Each mother and baby will be followed-up after birth by conducting four home visits (HV) over a period of 4 months. The first home visit (HV1) will be done when the infant is aged 2–3 weeks, with subsequent visits at 6–8 weeks (HV2), 12–14 weeks (HV3) and 14–18 weeks (HV4). The first three HV will be done in the morning (around 10–11 am) in order to maintain consistency in data collection, especially to maintain standardised procedures for the collection of breast milk and saliva samples. Mothers will be allowed to drink throughout the home visit session, but they will be advised not to eat, thus, they will be advised to have a meal prior to the session. Each home visit will take about 2–3 h and all sessions will be carried out by the same researcher. All mothers will be given a list contacts of BF peer counsellors and the nearest government clinics if they need any help with BF or have any concern regarding their infant health. During every home visit, all mothers will receive guidance and pamphlets about breastfeeding and infant development according to their infant’s age of stage of lactation.

##### Home visit I (HV1)

During HV1, the mother will be informed about the procedure for the visit and will be given a leaflet as a guide to the overall plan of the session. The mother will also be provided with a manual breast pump (Philips Avent brand) as a tool to express her breast milk sample if she wishes to use it. Mothers will also be shown how to use breast massage and hand expression techniques, which will allow her to choose the method she uses to express milk based on her preferences. Overall, during the first home visit, data will be collected from each mother-infant dyad as follows:

1. Collection of data on birth experience and initial breastfeeding practice using the CDC Neonatal Questionnaire.
2. Collection of data on breastfeeding practices and behaviour using an Infant Feeding Questionnaire (IFQ I), adapted from CDC and UK Infant Feeding Study 2010.
3. Collection of data on infant appetite using the Baby Eating Behaviour Questionnaire (BEBQ).
4. Infant weight, length and head circumference and maternal weight.
5. Mothers randomised to the relaxation group will be provided with an mp3 with the relaxation therapy recording to use during the breastfeeding session. The purpose of the exercises and imagery will be explained to the mother. Details of the therapy are described below.
6. Mini-breastfeeding test: The mother will be asked to collect samples of breast milk and saliva before and after a feed during the home visit without the presence of the researcher in the room. Mothers in the intervention group will be asked to listen to the relaxation/imagery recordings during this breast-feed. Prior to the feed, the mother will be asked (i) to collect a sample of her saliva to measure cortisol; (ii) to collect a sample of fore milk to measure cortisol, leptin, ghrelin and macronutrient content; (iii) to complete a questionnaire describing her feelings and emotions (Mini-breastfeeding Questionnaire). She will be asked to repeat these measures after completing the feed, including providing a sample of hind-milk; and to record the time and length of the feed. Mother’s saliva will be collected, and time and date will be recorded.
7. The mother will be dosed with deuterium to measure infant breast milk intake using the stable isotope method. The mother will be given written and oral instructions on collecting her saliva and urine samples from the infant post-dose.
8. The mother will be given a 3-day infant behaviour diary with instructions to complete after the visit; this will be collected at the next home visit.
9. At the end of the first home visit, mothers will be given a set of questionnaires to assess maternal psychological state (Edinburgh Postnatal Depression Scale (EPDS; depression), Perceived Stress Scale (PSS; stress), Beck Anxiety Inventory (BAI; anxiety) to complete at their convenience; these will be collected at the next home visit.

There will be no fixed order of measurement or assessment during the home visit session, but it will be based on the infant’s behaviour and needs. If the infant is hungry and/or is expecting a feed, the anthropometric assessment and mini-breastfeeding test will be done first and followed up with the questionnaire-led interview. Alternatively, if the baby is sleeping at the beginning of the session, the data collection from the questionnaires will be performed first. At the end of the visit, the mother will be provided with a folder of questionnaires and a diary calendar containing the isotope sample collection schedule. Following the visit, text messages will be sent to the mother as a reminder to collect isotope samples on day 1, 3, 4, 13 and 14 post home visit.

##### Home visit 2 (HV2)

The same measurements and data will be collected without the baseline isotope measurements since the infant breast milk intake will not be measured. Data on breastfeeding will be collected using the IFQ II questionnaire which contains different questions appropriate to the infant’s age and stage of breastfeeding.

##### Home visit 3 & 4 (HV3 & HV4)

Measurements and data collected at HV2 visit will be repeated during HV3, with a modified breastfeeding questionnaire (IFQ III). A repeat measurement of infant breast milk intake by isotope (dose to mother) will be done to measure the milk intake at 12–14 weeks of the infant’s age. At HV4, infant temperament will be assessed using the Revised-Rothbart’s Infant Behaviour Questionnaire (RIBQ). At this visit, measurement of infant body composition will be performed using the stable isotope method to measure total body water, and hence fat mass and fat-free mass.

### Intervention tool: The relaxation therapy recording

Mothers in the intervention group will be provided with a relaxation therapy tape to be used during breastfeeding or when they express milk. The tape recording consists of a modified guided imagery protocol based on a CD designed for breastfeeding mothers [16], which has been used previously in a study of mothers with preterm infants [13, 14]. The recording was transcribed and translated into the Malay language, in collaboration with a certified clinical psychologist in UPM (Dr Mukhtar). Mothers in the intervention group will be asked to listen to an audio recording with relaxation therapy (either Malay- or English-version according to their preference) while breastfeeding during every home visit sessions (HV1–3). They will be informed that the purpose of listening to the tape is to make the mother relax during breastfeeding and that this may or may not have beneficial effects on breastfeeding outcomes, which is one of the aims of the study. They will also be asked to listen to the recording daily while breastfeeding or expressing milk for at least 2 weeks starting at HV1 and again at HV2 and HV3. In between visits (from HV1 to HV3), they will be advised to keep using the relaxation therapy daily, as often as they find it useful, and will be given a calendar diary to record when it is used.

### Pilot study

After the relaxation recording was developed and translated into Malay, a pilot study was performed among breastfeeding women (*n* = 20) to investigate the overall perception towards the Malay-version of the recording by evaluating the voice, pace and intonation of the recording on a scale from 1 (strongly dislike) to 5 (strongly like). The participant’s emotions and feelings were also assessed after listening to the therapy by using a questionnaire that consisted of 7 items relating to emotional state on a 10 cm scale with the lowest scale (0) being ‘very little’ and the highest (10) being ‘very much’. The majority of participants liked the voice, intonation and pace of the relaxation therapy, with an average score of 83%, 78% and 67% respectively indicating ‘like’ to ‘strongly like’. Other participants indicated ‘neutral’ and only one person disliked each of the criteria. The top three scores for the assessment of emotion and feelings were awarded for ‘relaxed’(7.9), ‘happy’(7.8) and ‘alert’(7.6), and the lowest were ‘anxious’(1.1) and ‘stressed’(1.0). Thus, overall, the findings showed that the majority of participants in the pilot study had a good perception of the therapy, and it appeared to produce the expected effects on their emotions and feelings. A minor amendment to the voice and content of the Malay-version of the therapy was done to improve the recording based on feedback from the pilot study. A feasibility study was also done among several mothers (*n* = 5), prior to data collection, which helped the researcher to become familiar with the practical procedures and organize the home visit sessions effectively.

### Questionnaires

Several questionnaires will be used and repeated at each home visit, whilst a few will be used only at a specific time point. Below is the list of instruments that will be used in the study according to the time point, including those that will be used during enrolment (Table 2). Most questionnaires are available in both languages (English and Malay) to suit the Malaysian population. Several questionnaires (IIFAS, EPDS, PSS and BAI) have already been translated into the Malay language and have been used among mothers in Malaysia (the validity of the Malay-version of these questionnaires having been tested previously [17–19]**.** Those that are not translated into Malay will be performed using the interviewer-led questionnaire method, allowing standardisation of words used during the interview in all mothers. The overall questionnaires can be categorised into three domains; i) breastfeeding practices and attitudes, ii) infant feeding behaviour and temperament, and iii) maternal psychological state.

**Table 2:** List of data collection instruments that will be used in the MOM Study

No	Questionnaires	Language		Type of Question-naire^b^	Stages	Method of administration^c^
		English	Malay			
1	Socio-demographic Questionnaire	X	X	-	^a^post-enrolment	Self-completed
Breastfeeding practice and attitude:						
2	Iowa Infant Feeding Attitude Scale (IIFAS)	X	X	V	^a^post-enrolment	Self-completed
3	IIFAS Add Questionnaire	X	X	A	^a^post-enrolment	Self-completed
4	CDC Neonatal Questionnaire	X	X	A	HV1	Self-completed
5	CDC Infant Feeding Questionnaire I, II & III	X		A	HV 1,2,3	Interviewer led-questionnaire
Infant behaviour:						
6	Baby Eating Behaviour Questionnaire	X	X	V	HV 1,2,3	Self-completed
7	3-day Infant Behaviour Diary	X	X	V	HV 1 & 2	Self-completed
8	Revised-Rothbart’s Infant Behaviour Questionnaire (R-IBQ)	X	X	V	HV 4	Interviewer led-questionnaire
Maternal psychological state and emotion:						
9	Mini-breastfeeding Questionnaire	X	X	A	HV 1,2,3	Self-completed
10	Perceived Stress Scale	X	X	V	HV 1,2,3	Self-completed
11	Beck Anxiety Inventory	X	X	V	HV 1,2,3	Self-completed
12	Edinburgh Postnatal Depression Scale	X	X	V	HV 1,2,3	Self-completed

^a^Post-enrolment was done immediately after enrolment at the antenatal clinic

^b^V = Questionnaires that have been validated previously; A = Questionnaire that was adapted from previous studies;

^c^Self-completed = completed by the mother; Administered = Questionnaire-led interview by the researcher

### Socio-demographic questionnaire

This questionnaire will be self-completed by participants to determine their demographic background such as age, ethnicity, education levels, current or most recent occupation, household income and maternal birth order. The questionnaire also comprises questions on maternity care and traditional postpartum practices in Malaysia.

### Questionnaires on breastfeeding practice and attitude

Perception towards infant feeding and the mother’s goal for breastfeeding will be assessed when the participants are still pregnant using the Iowa Infant Feeding Attitude Scale (IIFAS), completed straight after enrolment at the antenatal clinic [20]. Data on birth experience and initial breastfeeding experience will be collected postnatally using the CDC Neonatal questionnaire during HV1. Interviewer-led questionnaires will be completed at HV1, 2 and 3 to assess breastfeeding practice and attitude at different stages of breastfeeding using the CDC Infant Feeding Questionnaire I, II and III [21].

#### Iowa infant feeding attitude scale (IIFAS)

The questionnaire consists of 17 questions and the participants will be asked to give their opinion and perceptions of infant feeding based on a scale of 1 (strongly disagree) to 5 (strongly agree). This questionnaire has been used extensively and has been tested for reliability with Cronbach’s alpha ranging from 0.85 to 0.86 [20]. There are a few questions added at the end of this questionnaire (IIFAS Add Questionnaire), which enquire about the mother’s response to a few statements on breastfeeding and their goal for feeding their infant after birth.

#### CDC neonatal questionnaire

This questionnaire is adapted from a cohort study in the US, the Infant Feeding Practices Study II, developed by the US Food and Drug Administration and the Centers for Disease Control and Prevention (CDC) [21]. Most questions are taken from the first cohort (Infant Feeding Practices I) and were tested in four pilot studies before being used in the survey. The adapted version of this questionnaire consists of three parts: source of information about breastfeeding; experience of birth and timing of first breast feed.

#### CDC infant feeding questionnaires (IFQ)

These questionnaires are adapted from the IFPS II and have been used in the First-Feed study among breastfeeding women (*n* = 50) in a sample of mothers in Glasgow, UK [22]. These questionnaires consist of 5 main parts: a) breastfeeding at present, b) breastfeeding in future, c) breastfeeding attitudes and difficulties, d) sleeping arrangements and e) health information. There are three different versions of the questionnaires: IFQ I, II and III, each suited to the infant’s age and stage of breastfeeding. These questionnaires are only available in English, therefore they will be completed through a questionnaire-led interview during each home visit (HV1–3).

### Questionnaires on infant behaviour

Infant feeding pattern and appetite will be assessed at different ages by the self-completed Baby Eating Behaviour questionnaire (BEBQ) at home visit 1–3 [23]. After home visits 1 and 2, participants will also be asked to record the duration of their infant’s feeding, crying and sleeping for three consecutive days in a 3-day infant diary [24]. Later, at the last home visit (HV4), infant temperament will be assessed using a Revised-Rothbart’s Infant Behaviour Questionnaire (RIBQ) [25].

#### Baby eating behaviour questionnaire (BEBQ)

The BEBQ is derived from an existing psychometric measure validated for older ages, the Children’s Eating Behaviour Questionnaire, supplemented by a review of the literature on milk-feeding behaviours. It has been used in a large birth cohort study in the UK (*n* = 4804), and appears to be reliable, with Cronbach’s alpha values ranging from 0.73 to 0.81 [23]. BEBQ can be used to measure infant appetite and eating behaviour during the period of exclusive milk feeding, which makes it well-suited for the new-born infant. It consists of 18 items designed to measure four traits, including a single item for general appetite (GE): ‘enjoyment of food’ (4 items), ‘food responsiveness’ (6 items), ‘slowness in eating’ (4 items), and ‘satiety responsiveness’ (3 items). The mothers will be asked to respond according to how they would described their infant feeding rate during a typical daytime feed based on a scale from 1 (never) to 5 (always).

#### 3-day Infant behaviour diary

Infant feeding and crying behaviour will be recorded at 2–4 and 6–8 weeks using a validated 3-day diary. The diary consists of a time scale for 72 h, which is divided into 15 min segments, and has five categories of behaviour: sleeping, crying, fussy, awake and content, and feeding [24]. Each category has its own characteristic shading pattern, and the mother will be asked to fill in the timescale with the appropriate shading according to the infant’s behaviour. The crying element has been validated using audio recordings [26].

#### Revised rothbart’s infant behaviour questionnaire (R-IBQ)

Development of temperament in the infant is rapid, varies across infancy, and is reliably observed starting at the age of 2 months [25]. Thus, infant temperament will be measured at 14–16 weeks using the validated RIBQ based on a 7-point Likert scale, from 1 (never) to 7 (always). Three major dimensions are used for the assessment of infant temperament: surgency/extraversion, negative affectivity and orienting/regulation. The reliability and validity of this questionnaire has been reported in many previous studies [25].

### Maternal psychological assessment

Maternal feelings and emotions before and after feeding will be assessed during the breastfeeding session of home visits 1, 2 and 3 using a Mini-breastfeeding questionnaire. After home visit sessions (HV1–3), participants will be given a set of questionnaires to be completed at their convenience, which were used to assess maternal stress, anxiety and depression using the Perceived Stress Scale, Beck Anxiety Inventory and Edinburgh Postnatal Depression Scale, respectively. The mother can answer this questionnaire in private.

#### Mini-breastfeeding questionnaire

The Mini-breastfeeding Questionnaire is a self-reported questionnaire that consists of 10-items used to measure the mother’s emotional state on a 10 cm scale, with the least being ‘very little’ and the most being ‘very much’. Mothers will be asked to record their feelings and emotions before and after each breastfeeding session during home visit 1–3. A vernier calliper will be used to measure the score that the mother marks on the scale.

#### Perceived stress scale

Cohen’s Perceived Stress Scale (PSS) is a psychological self-reported instrument that consists of 14-items for measuring the perception of stress on a scale of five, from 0 (never) to 4 (very often) [27]. It appears to be reliable, and has been validated and used extensively globally, including in three national surveys in the US [27, 28]. This questionnaire has also been translated and validated for the Malaysian population [19]. Each mother will be given both English and Malay version to answer after home visit 1–3, at their convenience.

#### Beck anxiety inventory

Beck Anxiety Inventory (BAI) is a self-reported instrument, consisting of 21-items, that is used to measure the severity of different aspects of anxiety such as numbness, fear, anxiety and nervousness, on a scale of four, from 0 (not at all) to 3 (severe). It has been proved to have high internal consistency and reliability (Cronbach’ss alpha values = 0.94) [29], and this has also been translated into Malay and validated in the Malaysian population with excellent overall alpha values (0.91) [17]. Each mother will be given both English and Malay version to answer after home visit 1–3, at their convenient.

#### Edinburgh postnatal depression

Edinburgh Postnatal Depression Scale (EPDS) is a self-reported questionnaire, consisting of 10-items questions that is used to screen and identify women with perinatal depression [30]. This questionnaire has been used extensively worldwide, is well-validated and appears to be reliable and sensitive in detecting depression [31]. This questionnaire has also been translated and validated for the Malaysian population [18]. Each mother will be given both English and Malay version to answer after home visit 1–3, at their convenience. When this questionnaire has been collected after each home visit session, the questionnaire’s score will be calculated to identify if the mother is depressed. If a mother is found to be depressed, she will be advised to seek help from a health professional.

### Anthropometric assessment

#### Measurements on mothers

Weight will be measured on a clinical weighing scale (Seca Meter, Germany) to the nearest 0.1 kg at all home visits. Each measurement will be repeated three times. The mother’s height and pre-pregnancy and late gestation weight will be recorded based on the latest antenatal clinic’s report.

#### Measurements on infant

Anthropometric measures (recumbent length, weight and head circumference) on the infant will be carried out on all home visits. All measurements will be repeated three times and the mean value will be used. The BMI will also be calculated from the anthropometric data.

#### Infant weight

Weight will be measured on a digital infant weighing machine (brand Seca 834) prior to the breastfeeding session. The weighing machine will be calibrated regularly throughout the data collection period. During the measurement, a towel will be placed over the scale before resetting to zero. All infants will be weighed naked, with an accuracy of 0.01 kg.

#### Infant recumbent length

Infant length will be measured to the nearest 1 mm using an Infant Length Measuring Mat (Rollameter 60, UK) with a fixed headboard at one end and a measuring tape on a movable vertical plate at the other end. During the measurement, the infant will be supine, with the head against the fixed headboard and the body parallel to the board’s axis, following the Frankfurt Plane position. The vertical plate will be placed against the base of the infant’s feet. The infant’s legs will be straightened by holding the ankles with one hand and applying a gentle downward pressure over the legs with the other hand. The mother will be asked to check if the infant’s head is still in position and touching the headpiece. Once the infant is in a straight line position with feet straight, the vertical plate will be mounted to touch the soles of the infant’s feet, with toes pointing directly upward. The measurement will be read from the red arrow in the reader window.

#### Head circumference

A flexible non-stretchable measuring tape (SECA 212, Germany) will be placed just above the eyebrows and ears, and around the occipital prominence at the back of the head, so that the maximum circumference (largest diameter) is measured. The measurement will be read to the nearest mm.

#### Infant body composition

Infant body composition will be measured using the isotope dilution method at the final home visit (HV4) when the infant is aged 14–18 weeks. This method is based on the assumption that fat-free mass has a relatively constant water content with insignificant water associated with fat stored in adipose tissue [32]. To perform this measurement, the deuterium oxide dilution (^2^H_2_O) will be administered orally by dripping the dilution directly into the infant’s mouth using a 10 ml syringe, as the previous study showed this to elicit the best cooperation from the infants [33]. Some infants will be administered milk using a sterilised bottle depending on the mother’s preferences. Weighed tissues will be placed near the baby’s mouth while dosing to wipe any spillage. After dosing, the syringe or milk bottle and used tissues will be re-weighed to estimate the exact amount of dose that is given to the baby. Infant urine samples will be collected at baseline (prior to dose) and following 24- and 48-h post-dose. All samples collected will be stored in the freezer at −80 °C until analysis. The isotope enrichment samples will be measured by isotope ratio mass spectrometry [32, 34].

### Collection of biological samples

During HV1 to HV3, mothers will be asked to provide samples of breast milk and saliva before and after a BF session for measurement of cortisol levels. Breast milk samples will also be used to measure the macronutrient levels. Maternal saliva and infant urine samples will be collected to measure breast milk intake using the isotope dilution method. All samples collected will be kept cool in an insulated box containing frozen silica pads, before being stored in the freezer within 4 h after collection.

#### Collection of breast milk samples

The mother will be asked to express about 10–15 ml of breast milk before and after breastfeeding session, with the time of collection being recorded. A manual breast pump (Philips Natural, UK) will be provided to each mother, with written and oral instructions provided. The mother will be given the option to express milk either by hand or using the pump provided, or their own pump, depending on their preferences. Prior to expressing milk, all mothers will be encouraged to massage their breast in order to stimulate milk ejection. Milk samples will be stored temporarily in milk storage containers, which will be kept in an insulated box containing a frozen silica pad during the visit. After completion of the home visit, milk samples will be transferred into 15 ml tubes and a portion of the samples will be acidified and transferred into 2 ml tubes. All samples will be stored at −80°C until analysis.

#### Collection of saliva samples

The mother will be asked not to eat or drink for at least half an hour prior to sample collection. The saliva sample will be collected using a salivary oral swab (salivette) (Salimetrics, UK), which the mother gently rolls in her mouth for 2 min before placing into a salivette tube. After home visits, salivettes will be centrifuged and saliva samples will be transferred into 2 ml tubes, which then will be stored at −80°C until analysis.

#### Collection of isotope samples

Milk volume will be measured at 2–4 week (as baseline data at HV1) and 12–14 week (at HV3) using stable isotopes which allow the measurements to be performed without interfering with the breastfeeding process [32, 34]. To dose the mother, she will be asked to drink approximately 30 g deuterium dilution (^2^H_2_O) through a straw. A maternal baseline salivary sample will be obtained by using a salivette prior to dosing and during the following 14 days post-dose (day 1, 4 and 14). The mother will be asked not to eat or drink for 30 min prior to collecting a saliva sample. Infant urine samples will also be collected at day 0 (baseline sample) and then over 14 days post-dose (day 1, 3, 4, 13 and 14). Detailed instructions on collection of urine samples will be provided and demonstrated to the mother during the first home visit session. To estimate infant total body water, additional urine samples will be collected after administering 10 g deuterium (^2^H_2_O) to the infant as indicated above; baseline urine and post-dose samples will be collected after 24- and 48 h. All samples will be stored in 2 ml tubes at −80°C until analysis. Total milk intake and total body water will be estimated based on the measurement of deuterium enrichment by isotope ratio mass spectrometry (IRMS) [32, 34].

#### Analysis of biological samples

Breast milk samples will be analysed to determine the hormonal (cortisol, leptin and ghrelin) and macronutrient content. The macronutrient content of breast milk will be analysed using the MIRIS analyser at UCL. The breast milk cortisol, leptin & ghrelin will be measured by commercially available kits for enzyme-linked immunosorbent assay (ELISA) using cortisol saliva human ELISA, leptin human ELISA and ghrelin acylated & total human ELISA kits, respectively.

### Plan of statistical analysis

Data that are later found to be ambiguous will be flagged up and re-checked, and mothers will be contacted for clarification where necessary. Data will be recorded initially in Excel datasheets and later exported to SPSS. All questionnaires and anthropometric data will be analysed using IBM SPSS (version 23). Normality of continuous data will be assessed by Q-Q Plot, histogram and will also be tested by the Kolmogorov-Smirnov Test. The main analysis will be an intention-to-treat analysis comparing the difference between groups in the final outcome adjusted for the baseline value where data are available using independent t-tests. Independent t-test and repeated measures will be used to examine changes in milk intake and other milk components including cortisol levels between groups and between time points respectively as secondary analyses. For the secondary outcomes, associations between infant temperament/behaviour and milk volume, milk composition, infant growth and body composition will be examined using univariate analyses. Correlation and regression analysis/MANOVA will then be used to adjust for confounding factors. Interaction terms will be introduced where appropriate, especially in the multivariate analysis. The cut-off used for significance will be set at *p* < 0.05. Although multiple testing will be done to test the outcomes, the *p*-value cut-off point will not be adjusted for multiplicity since the different outcome measures are independent of each other.

Anthropometric data will be converted to standard deviation score (SDS, Z-score) for infant weight, height, head circumference and BMI using WHO 2006 standard data (LMS growth add- in for Microsoft Excel) and SDS will be used in all analyses. The weight data at the final time point will be compared between groups and adjusted for baseline weight as the primary outcome measure for growth using the LMS ‘Weight gain to SDS’ based on the WHO 2006 growth standard data (ie. external data). This method takes into account the baseline weight (HV1) in calculating the change from baseline to an endpoint or increase in weight between two-points of measurements. An increment of more than 1 band on the WHO growth chart or ±0.67 SD between measurements will be considered as representing ‘rapid weight gain’ [35].

## Discussion

The majority of the population in Malaysia, especially first-time mothers, practice a traditional postpartum confinement period for about 30–45 days, according to the cultural tradition of their ethnic background [36]. During this period, the mother and her infant will be taken care of either by their family members, particularly the infant’s grandmother, or by a confinement lady who will be paid to stay overnight with the mother or come to the house on a daily basis. The caretaker will usually prepare meals for breakfast, lunch and dinner following the cultural confinement diet for the mothers, and will also perform special postpartum traditional massage on the mother, especially during the first 3 weeks of the postnatal period [36]. The caretaker will also help to take care of the infant by changing the nappy, bathing or holding the infant when necessary. This is because the mother is encouraged to minimise physical activity and have ample rest to recover from the birth [36]. This confinement practice should facilitate the trial since all mothers in the study will receive similar care.

The composition of human milk varies across lactation, and between and within individuals diurnally and across a single feeding. Thus, milk sampling is an important issue, as there is a lack of consistency in sample collection in previous studies [37, 38]. One possible explanation for the inconsistent findings between published studies is the different methods used for sample collection (e.g. single spot sample or pooled milk sampling), as well as a lack of standardisation of timing with respect to both time of day or stage of lactation [37, 38]. This is especially important when measuring milk energy density in breast milk, as the changing fat content between fore- and hindmilk affects the total energy content. Presumably, this will also influence the content of other fat-soluble substances in breast milk, such as fat-soluble vitamins or other bioactive substances. To date, there is no universal sampling protocol or gold standard method for human milk sampling since there are various ethical aspects to be considered as well as limitations of the population and conditions in the field [38]. Thus, comparing data from different studies is challenging and problematic, especially for data that are based on a single milk sample collection. Hence, in this trial, fore- and hindmilk will be collected at 3 different time points and the milk sampling protocol will be standardised in terms of time and sampling method in order to minimise the variability. Moreover, unstandardized sample collection methods and different times for sampling may lead to unrepresentative samples being used during analysis. Therefore, in this trial, the isotope method will be used to estimate milk intake over a period of 14 days, which has the advantage of being physiological (ie. suckled breast milk), non-invasive (since it does not interfere with the breastfeeding process), and suited to free-living infants [32, 34].

In summary, the experimental design of this study aims to show causal relationships between maternal psychological state and infant behaviour and growth, which may be mediated through breast milk composition and/or volume. By investigating the mechanisms of biological and behavioural signalling during early human life, this project aims to provide a greater understanding of maternal-infant factors which influence BF outcomes. This may in turn allow identification of modifiable factors that can be useful targets for future interventions to increase breastfeeding duration.

## Abbreviations

BAI: Beck Anxiety Inventory
BEBQ: Baby Eating Behaviour Questionnaire
BF: Breastfeeding
EDD: Estimated delivery date
ELISA: enzyme-linked immunosorbent assay
EPDS: Edinburgh Postnatal Depression Scale
HV: Home visit
IFQ: Infant Feeding Questionnaire
IIFAS: Iowa Infant Feeding Attitude Scale
MANOVA: Multivariate analysis of variance
MOMS: Mother-Offspring Milk study
PSS: Perceived Stress Scale
RIBQ: Revised-Rothbart’s Infant Behaviour Questionnaire
